# Factors Associated with Acute Respiratory Infections in Children Under Five Years Old: Analysis of the Demographic and Family Health Survey

**DOI:** 10.3390/children12091242

**Published:** 2025-09-16

**Authors:** Diego A. Polo-Pucho, Javier J. Gonzales-Carrillo, Miguel A. Arce-Huamani

**Affiliations:** 1 Facultad de Ciencias de la Salud, Universidad Privada Norbert Wiener, Lima 15046, Peru; diegopolopucho@gmail.com (D.A.P.-P.); javier.gonzales@uwiener.edu.pe (J.J.G.-C.); 2 Vicerrectorado de Investigación, Universidad Privada Norbert Wiener, Lima 15046, Peru

**Keywords:** good health and well-being, respiratory tract infections, child, epidemiology, demographic and health surveys, Peru

## Abstract

**Background/Objectives:** Acute respiratory infections (ARIs) remain a leading cause of morbidity and mortality in children under five in low- and middle-income countries. This study aimed to estimate the prevalence of caregiver-reported ARI symptoms and identify independent risk factors among Peruvian children under five using a nationally representative survey. **Methods:** We conducted an analytical cross-sectional study using the 2022 Demographic and Family Health Survey (ENDES) of Peru. Children under five with complete data were included. ARI symptoms were defined from the standardized DHS/ENDES question asking whether the child had a cough in the preceding two weeks. Analyses accounted for the complex survey design (weights, strata, primary sampling units). Associations were evaluated using modified Poisson regression with robust standard errors to estimate prevalence ratios (PRs) and 95% confidence intervals (CIs). **Results:** Among 6299 children under five, the prevalence of caregiver-reported ARI symptoms was 38.5%. After multivariable adjustment, male sex (adjusted PR: 1.07; 95% CI: 1.00–1.14; *p* = 0.04) and age 1 to <3 years (adjusted PR: 1.18; 95% CI: 1.09–1.27; *p* < 0.001) and 3 to <5 years (adjusted PR: 1.14; 95% CI: 1.05–1.24; *p* = 0.001) were associated with higher prevalence compared with infants <1 year. Wealth quintile, maternal education, and low birth weight were not independently associated with ARI symptoms. **Conclusions:** Caregiver-reported ARI symptoms remain highly prevalent among Peruvian children under five. Sex- and age-specific differences highlight the need for context-sensitive prevention, caregiver education, and efficient resource allocation within nationally representative, survey-informed child-health strategies.

## 1. Introduction

Acute respiratory infections (ARIs) remain a leading cause of morbidity in children under five across low- and middle-income countries, with patterns that vary by age, sex, sanitation, and urban–rural residence [1,2,3]. In Sub-Saharan Africa, the prevalence of acute lower respiratory tract infections in children under five varies widely, ranging from 1.9% to 60.2%, with risk heightened by factors such as poor education, poverty, malnutrition, exposure to smoke, and inadequate sanitation [1]. National analyses have shown that the use of contaminated fuels for cooking is significantly associated with ARI risk, with children in such households being 69% more likely to experience an ARI episode compared to those using clean fuels [4]. Additionally, both individual and contextual determinants such as maternal education, household wealth, and urban residence have demonstrated strong associations with ARI prevalence in large-scale studies across 37 Sub-Saharan African countries [2]. These patterns highlight the complexity and persistence of ARIs as a public health concern in young children [5].

Despite advances in reducing overall under-five mortality, substantial gaps persist in understanding the regional and local variations in ARI burden and the specific risk factors that drive this heterogeneity, especially within countries characterized by diverse populations and health systems [6,7]. Recent evidence from South Asia and Africa emphasizes the need to consider a broader set of determinants, including child age, maternal smoking, household sanitation, and nutritional status, yet existing studies have often lacked granularity in evaluating how these factors interact or differ by context [8]. Facility-based studies continue to report substantial clinical burden in specific settings, while evidence remains limited on the relative contribution of coexisting conditions such as malnutrition and anemia to ARI risk across diverse contexts [9].

Furthermore, community-based studies in East Africa have highlighted that common clinical symptoms and outpatient diagnostic challenges can lead to misdiagnosis or delayed treatment, underscoring the need for better epidemiological characterization and health system responses in resource-limited environments [10]. Additionally, cross-national reviews have pointed to variations in ARI determinants such as diarrhea comorbidity, maternal education, and household socioeconomic status, but few studies have comprehensively analyzed these factors using nationally representative survey data in Latin America or have compared their effects across urban and rural contexts [1,11]. Thus, a key gap remains in disentangling the multifactorial drivers of ARI at both the individual and community levels, especially in countries with high demographic diversity. In this context, studies leveraging large, representative datasets and robust analytical frameworks are urgently needed to inform targeted interventions and policies.

Using the nationally representative 2022 Demographic and Family Health Survey (ENDES), we estimate the prevalence of caregiver-reported acute respiratory infection (ARI) symptoms among children under five and identify independent risk factors while accounting for the survey’s complex, multistage design. Our aim is to deliver robust, policy-relevant evidence to inform targeted child respiratory-health interventions in Peru.

Despite sustained declines in under-five mortality, acute respiratory infection (ARI) remains a leading infectious cause of child deaths globally and disproportionately affects children in low- and middle-income countries. Recent WHO estimates indicate that pneumonia alone killed roughly 740,000 children under five in 2019, with the highest mortality concentrated in sub-Saharan Africa and South Asia, evidence of a persistent, unequal burden that demands renewed policy attention (WHO, 2022) [12]. Beyond mortality, nationally representative DHS/MICS analyses reveal substantial between-country heterogeneity in caregiver-reported ARI symptoms and in their social and environmental determinants, reinforcing the need for current, country-specific evidence built on comparable methods (e.g., multilevel, pooled analyses across many LMICs) [13].

For Peru, up-to-date, nationally representative multivariable analyses that jointly evaluate demographic, social, and environmental determinants of ARI using standardized operational definitions and complex-survey estimators are scarce. Given known measurement limitations in household surveys (caregiver-reported symptoms, two-week recall, and definitional differences across DHS/MICS waves), transparent handling of misclassification and design features is essential. An ENDES-based assessment that (i) applies harmonized coding, (ii) accounts for weighting, stratification, and clustering, and (iii) quantifies independent associations with policy-relevant factors is needed to guide targeted prevention and resource allocation in Peru [13,14].

This analytical, cross-sectional study uses nationally representative 2022 ENDES data to estimate the two-week prevalence of caregiver-reported acute respiratory infection (ARI) symptoms among Peruvian children under five and quantify independent associations with demographic, household, and contextual factors using modified Poisson regression under the complex survey design [15]. Applying harmonized DHS/ENDES operational definitions and transparent handling of measurement limits, our goal is to deliver policy-relevant, design-based estimates that can guide targeted prevention and efficient resource allocation in Peru, considering the persistent global ARI burden in under-fives [13,14,15].

This study provides design-based, nationally representative evidence for Peru using ENDES 2022 and a transparent, standardized operationalization of ARI aligned with DHS/ENDES conventions. By estimating independent associations between ARI symptoms and policy-relevant determinants (child age/sex, household and contextual factors) via modified Poisson regression under the complex survey design, our findings are immediately actionable for Peruvian stakeholders (e.g., health authorities, pediatric providers, immunization and primary-care programs). The approach enhances replicability (clear coding, predefined covariates), comparability with other DHS/MICS settings, and translation into practice (risk stratification, targeted caregiver education, region-specific prevention). In doing so, it addresses a current evidence gap: recent, national, multivariable analyses of ARI in Peru that explicitly handle measurement limits in caregiver-reported symptoms while correctly accounting for weighting, stratification, and clustering [16].

This study is guided by the Social Determinants of Health (SDOH) framework [17], which posits that child health reflects broader social and environmental conditions (e.g., caregiver education, sanitation, household crowding, urban–rural residence, and regional context). To interpret care-seeking and caregiver reporting of symptoms, we also draw on the Health Belief Model (HBM) [18] highlighting perceived severity and susceptibility, perceived barriers (e.g., travel time, costs), and cues to action. Together, SDOH and HBM inform our a priori covariate selection and support a structured interpretation of observed ARI patterns in Peru.

## 2. Materials and Methods

### 2.1. Study Design and Setting

This analytical, cross-sectional study used secondary data from the 2022 Demographic and Family Health Survey (ENDES), a nationally representative survey implemented by the National Institute of Statistics and Informatics (INEI) of Peru. The ENDES sample is two-stage, probabilistic, balanced, stratified, and independent at the departmental level (according to the country’s political–administrative division) and is stratified by urban and rural areas. In urban areas, the primary sampling units are clusters of contiguous private dwellings; in rural areas, they are rural census areas and private dwellings.

The unit of analysis comprised usual residents of selected private dwellings in both urban and rural areas, specifically those who spent the night prior to the survey in the selected household. Data were collected through face-to-face interviews administered by trained personnel using mobile devices (tablets), ensuring standardized, high-quality data acquisition. Further technical details regarding the sampling process and survey design of the 2022 ENDES are available in the official INEI documentation. This analysis focused on children under five years of age; fieldwork was conducted from January to December 2022.

Representativeness and nonresponse. ENDES is designed for national and subnational representativeness with nonresponse adjustments embedded in the sampling weights. Participation is voluntary; detailed response rates are reported by INEI in the technical documentation. Although survey weighting, stratification, and clustering were applied in all analyses, differential nonresponse by geography or socioeconomic status may persist; we, therefore, interpret nationally representative estimates with this caveat and explicitly acknowledge possible nonresponse bias in the Limitations.

### 2.2. Population and Sample

We included all de jure children aged 0–59 months residing in sampled households and interviewed in the child-health module of ENDES 2022. Records were excluded if the ARI outcome (H31; caregiver-reported cough in the past two weeks) or any a priori covariate was missing: child sex (B4); age in months (B8; recoded to <1, 1–<3, and 3–<5 years); area of residence (V102); household wealth quintile (HV270/HV271); mother’s education (V106); completed national immunization schedule (S45PV1, S45PV2, S45PV3, S45NM1, S45NM2, S45IF1, S45IF2, S45IF3, S45B0, S45F1, S45F2, S45ML, S45RT1, S45RT2, as applicable); low birth weight (M19); exclusive breastfeeding (QI440B; for infants 0–5 months); sanitation (HV205); overcrowding components (HV009, HV216); and child health insurance (S229B1). The final analytic sample comprised 6299 children. Table 1 presents survey-weighted distributions for all variables; model-specific sample sizes are shown in the footers of Table 2 and Table 3. Missing-data procedures are described in Statistical Analysis.

### 2.3. Data Collection

Data were collected by trained interviewers through standardized, face-to-face surveys using electronic tablets under INEI protocols. Fieldwork quality assurance included centralized supervision, periodic spot checks and back-checks, and built-in electronic consistency checks in the CAPI system. Interviewers received standardized training, and field procedures were monitored throughout January–December 2022 to ensure completeness and internal consistency.

### 2.4. Variables

The primary outcome was caregiver-reported acute respiratory infection (ARI) symptoms within a two-week recall period, based on standardized DHS/ENDES child-health questions. Specifically, caregivers were asked: “In the last two weeks, has the child had a cough?” No additional symptoms (e.g., fever or fast/short breathing) were required; this cough-based definition follows DHS/ENDES conventions to maximize cross-study comparability. We created a binary indicator (1 = caregiver-reported ARI symptoms; 0 = otherwise). Because this measure does not involve clinical examination, we refer to the outcome as ARI symptoms rather than physician-diagnosed ARI. Cough may also arise from non-infectious etiologies (e.g., reactive airways disease or allergic triggers) or upper-airway conditions, and symptom ascertainment depends on caregiver recognition and recall, which may occasionally be by a non-primary caregiver. Any resulting misclassification is likely non-differential with respect to sociodemographic covariates and would bias prevalence ratios toward the null.

#### Operational Definitions and Coding

Covariates followed DHS/ENDES conventions and were coded as follows: child sex (female/male); child age (under 1 year; 1 to <3 years; 3 to <5 years); area of residence (urban/rural); natural region (Coast/Andes/Amazon); household wealth quintile (ENDES quintiles 1–5); maternal education (none/primary/secondary/higher); overcrowding (≥3 persons per bedroom vs. <3); sanitation (improved vs. unimproved facilities per international standards); completion of the national immunization schedule (yes/no; in ENDES, recorded from the child’s vaccination card when available and otherwise from caregiver recall); low birth weight (<2500 g vs. ≥2500 g); exclusive breastfeeding (only breast milk for the first 6 months: yes/no); and child health insurance (enrolled in any public or private plan: yes/no). Original ENDES variable names and recording rules will be provided in Supplementary Table S1 to ensure full replicability.

### 2.5. Data Analysis

We estimated prevalence ratios using modified Poisson regression with robust (sandwich) standard errors [19], an approach that recent methodological work recommends for common binary outcomes in cross-sectional/cohort data and for which practical goodness-of-fit and implementation guidance are now available [20,21,22]. All models were fit under the complex survey design, incorporating sampling weights, strata, and primary sampling units (PSUs), with variance estimated via Taylor series linearization. Covariates were specified a priori based on recent literature and a Social Determinants of Health framework (child age and sex; maternal education; household wealth; sanitation; overcrowding; urban–rural residence; and natural region). Two-sided *p* < 0.05 was considered statistically significant.

Survey design specification. All analyses used Stata’s survey procedures with the complex design applied. We specified primary sampling units (PSU = v021), stratification (strata = v022), and sampling weights (pweight = v005/1,000,000), following DHS/ENDES conventions. Where household-level variables were merged, the corresponding household identifiers and weights (hv021/hv022/hv005) were used as appropriate. Variance estimation employed Taylor series linearization under the survey design. All analyses were conducted in Stata 18 (StataCorp, College Station, TX, USA).

Descriptive and bivariate analyses. We produced survey-weighted descriptive statistics (frequencies and percentages) for all variables. Bivariate associations between ARI symptoms and each covariate were examined using design-adjusted chi-square tests [23].

Model specification and covariate selection. Multivariable models were specified as a priori from recent literature and conceptual frameworks (Social Determinants of Health; Health Belief Model), not solely on bivariate *p*-values. The predefined covariate set (child age and sex; maternal education; household wealth; sanitation; overcrowding; urban–rural residence; and natural region) was retained regardless of bivariate significance to minimize residual confounding. Additional variables were considered when conceptually warranted and retained if they demonstrated independent associations (two-sided *p* < 0.05) without inflating multicollinearity [24].

Missing data handling. We conducted a complete case analysis. Responses coded as “don’t know/unknown” in ENDES were treated as missing and excluded from the multivariable model. Model sample sizes (N) are reported for each table. Because item-missingness can be differential by socioeconomic status or geography, we acknowledge the potential for selection bias from complete-case analysis in the Limitations.

Model diagnostics and robustness. We assessed multicollinearity using variance inflation factors (VIFs); all VIFs were <5. We examined model stability by comparing nested specifications that sequentially added a priori covariate blocks; estimates for the main associations (sex and age) were materially unchanged. We used robust (sandwich) standard errors under the complex survey design, inspected Pearson residual patterns, and found no evidence of model misspecification affecting inference.

### 2.6. Ethical Considerations

This research used publicly available, de-identified secondary data from the 2022 ENDES survey. Participation in ENDES was voluntary, and informed consent was obtained by the National Institute of Statistics and Informatics (INEI) at the time of data collection. All analyses adhered to the ethical principles of the Declaration of Helsinki and complied with applicable national regulations for the secondary use of public health survey data. The protocol was reviewed and approved by the Research Ethics Committee of Universidad Privada Norbert Wiener (Approval No. 0104-2024). The ENDES dataset is publicly available in de-identified form; no contact with human participants occurred, and no personally identifiable information was accessed.

### 2.7. Data Availability and Use of Generative AI

Publicly available ENDES 2022 microdata, questionnaires, and technical documentation are accessible through the INEI microdata portal; no special permissions are required. We analyzed de-identified records only, and no new data were created.

No generative artificial intelligence (AI) tools were used in the design, data collection, statistical analysis, or interpretation of this study.

## 3. Results

### 3.1. Descriptive Analysis of Results

Table 1 summarizes the sociodemographic and clinical characteristics of children under five years of age included in the 2022 ENDES survey. The sample comprised nearly equal proportions of females (49.2%) and males (50.8%), with most children residing in urban areas (68.3%). One third were under one year of age (33.7%), while 35.0% were aged one to less than three years, and 31.3% were three to less than five years old. Socioeconomic distribution showed a higher concentration in the first (30.5%) and second (29.1%) wealth quintiles. Most children (69.1%) had completed the national vaccination schedule, and most mothers reported secondary (52.5%) or higher (32.9%) education. Only 7.1% of children were born with low birth weight, while 95.4% received exclusive breastfeeding. Health insurance coverage was reported for 86.7% of the children. Using survey weights, the two-week prevalence of caregiver-reported ARI symptoms was 38.5%. Weighted prevalence varied by sex (higher among boys), across age groups (higher in 1 to <3 years and 3 to <5 years vs. <1 year), and by urban–rural residence and natural region. Stratified, design-based estimates are reported in Table 2; all percentages reflect the complex survey design.

### 3.2. Bivariate Analysis

Table 2 displays the design-based bivariate analysis of sociodemographic and clinical characteristics by ARI status. The sex distribution differed by ARI status (*p* = 0.04). Children aged 1 to <3 years represented a greater proportion among ARI cases versus non-cases (37.5% vs. 33.5%), while infants <1 year were less frequent among ARI cases (30.4% vs. 35.7%, *p* < 0.001). No significant differences were observed by area of residence (urban/rural, *p* = 0.29). Socioeconomic disparities were evident across wealth quintiles (*p* = 0.002). Completion of the national immunization schedule and health insurance coverage were similar between groups. Maternal education differed (higher secondary, lower higher education among ARI cases; *p* < 0.001). All descriptive and bivariate estimates are design-based and incorporate ENDES sampling weights, stratification, and clustering.

### 3.3. Multivariate Analysis

In multivariable modified Poisson regression, male sex (PR 1.07, 95% CI 1.00–1.14, *p* = 0.04) and older age groups remained independently associated with higher ARI prevalence (Table 3). Compared with infants <1 year, children 1 to <3 years (PR 1.18, 95% CI 1.09–1.27, *p* < 0.001) and 3 to <5 years (PR 1.14, 95% CI 1.05–1.24, *p* = 0.001) had higher prevalence. The inverse association observed for the highest wealth quintile in crude analysis attenuated and was not statistically significant after adjustment (PR 0.88, 95% CI 0.76–1.07, *p* = 0.08). Maternal education and low birth weight were not significantly associated with ARI in adjusted models.

Diagnostic checks indicated no problematic multicollinearity (all VIFs < 5), and the main estimates were stable across nested specifications; design-based robust standard errors yielded the same inferential conclusions.

## 4. Discussion

Our findings demonstrate that acute respiratory infection (ARI) remains a substantial health challenge for Peruvian children under five: in the nationally representative 2022 ENDES, approximately 38.5% of children had caregiver-reported ARI symptoms within a two-week window. The elevated risk observed among boys and among children aged 1 to <5 years likely reflects a combination of biological, immunological, and social factors. The observed male excess in ARI symptoms may reflect sex-specific differences in early immune maturation and epithelial receptor profiles; however, these pathways were not directly measured in ENDES and should be interpreted cautiously. Early childhood also entails increased exposure to environmental pathogens, greater interaction with peers in daycare or community settings, and an evolving yet still immature immune system. These patterns underscore persistent vulnerabilities in this population. Our results highlight the need for focused interventions, including proactive case detection, enhanced caregiver education, and tailored immunization and prevention strategies for the most at-risk groups. Addressing these gaps could reduce ARI-related morbidity and advance child health equity in Peru and other countries facing similar epidemiological challenges.

Biological rationale for male excess. The higher prevalence among boys is consistent with sex-specific differences reported in pediatric immunology, including earlier-life patterns of innate and adaptive immune maturation and variation in airway epithelial receptor expression for common respiratory pathogens. These mechanisms were not measured in ENDES; therefore, this interpretation is cautious, and anatomic differences alone are unlikely to be the principal driver of the observed sex gradient.

The prevalence of acute respiratory infection (ARI) observed in our study among Peruvian children under five years old was notably high. When comparing these findings to other national and international studies, important differences become apparent. For instance, a much lower prevalence was reported in Bangladesh (2.03%) using the MICS/BDHS definition of respiratory symptoms within the preceding two weeks [25], and similarly low rates were found in Indonesia (4.2% among children under five, IDHS 2017) [26]. Conversely, hospital-based studies in Ethiopia and Nepal reported substantially higher prevalence rates of 27.3% and 60.8%, respectively, likely reflecting the more severe or symptomatic cases captured in healthcare settings [27,28]. In Africa, Ghana’s national survey indicated a prevalence of 33.3% [29], while in Zambia the prevalence declined from 13% in 1996 to 4% in 2014 [30]. Notably, an alternative survey in Bangladesh, which used broader ARI definitions, reported rates comparable to our findings, with 39.27% in 1997 and 43.27% in 2014 [3]. These discrepancies can be attributed to differences in case definitions, data sources (community versus hospital-based), recall periods, and local epidemiological contexts. Understanding these variations is crucial for contextualizing ARI prevention strategies and for benchmarking progress towards reducing childhood respiratory infections globally.

Our study identified a higher risk of ARI among male children compared to females, aligning with the hypothesis that biological and behavioral differences may influence susceptibility. This finding is consistent with recent evidence from Bangladesh, where being female was associated with a significantly lower risk of ARI (AOR 0.68; 95% CI 0.56–0.83), confirming male sex as a risk factor in multivariate analysis [25]. However, not all studies corroborate this pattern. In Indonesia, no significant association between sex and ARI was observed in the bivariate analysis [26], while in Ethiopia, cases were distributed equally among boys and girls, with no statistically significant relationship reported [27]. Similarly, large-scale analyses in Bangladesh and Nepal either found no statistically significant differences by sex or did not provide adjusted results [3,28]. The variability in these findings may be attributed to differences in study design, sample size, and control for confounding factors, as well as sociocultural influences affecting healthcare-seeking behavior for boys and girls. Recognizing the nuanced role of sex in ARI risk is essential for tailoring public health interventions that ensure equity in the prevention and management of childhood respiratory infections.

In our analysis, children aged one to less than five years demonstrated a higher risk of ARI compared to infants under one year, suggesting that increasing age within early childhood is associated with greater exposure and vulnerability. However, this trend contrasts with several international studies, which predominantly report a higher risk of ARI among younger children. For example, in Bangladesh, children aged 0–23 months had a greater likelihood of ARI than those aged 24–59 months (AOR 0.53 for 24–59 months versus 0–23 months), indicating heightened vulnerability in infancy [25]. Similarly, in Ethiopia, infants under 12 months had a significantly higher risk than children over 48 months (AOR 3.39; 95% CI 1.19–9.65) [27], and in Zambia, the risk was also greater in children under one year [30]. On the other hand, studies from Indonesia and Ghana identified an elevated risk in children aged one or two years compared to newborns or infants, partially aligning with our findings [26,29]. These divergences likely reflect contextual factors such as varying exposures, breastfeeding practices, vaccination coverage, and access to care. Clarifying age-related risk patterns is vital for directing age-specific ARI prevention efforts, especially in resource-limited settings where the burden of respiratory infections remains substantial.

In our study, no significant association was found between wealth quintile and the risk of ARI in children under five, suggesting that socioeconomic status, as measured by household wealth, did not independently predict ARI in this context. This result is consistent with findings from Bangladesh, where multivariate analysis also showed no significant relationship between wealth quintile and ARI risk [25]. Similarly, the national survey in Ghana found no significant association between wealth status and ARI, although such a relationship was evident for other childhood conditions like diarrhea [29]. In contrast, studies in Indonesia and Bangladesh have reported that children from poorer households are at higher risk of ARI. For instance, the Indonesian analysis found that the poorest and poor quintiles had significantly greater odds of ARI compared to the richest group (OR 1.91, 95% CI 1.26–2.89 for poorest; OR 1.50, 95% CI 1.01–2.21 for poor) [26], while in Bangladesh, higher wealth was protective (OR for richest 0.824, 95% CI 0.701–0.968) [3]. These discrepancies may reflect differences in the distribution of risk factors across populations, the effectiveness of social protection and health interventions, or variations in health-seeking behaviors. Understanding the nuanced role of socioeconomic status is essential for developing equitable public health strategies to reduce ARI in diverse settings.

Our findings indicated no significant association between maternal education level and the risk of ARI in children under five, as confirmed in multivariate analysis. This result aligns with several studies from different contexts. For example, both Bangladesh and Indonesia reported no significant association between maternal education and ARI after adjusting for other factors [25,26]. Similarly, in Ghana, maternal education was not significantly related to ARI risk in multivariable analysis, although an association was observed for diarrheal disease [29]. However, evidence from Zambia suggested a protective effect of higher maternal education, with an adjusted odds ratio of 0.30 (95% CI 0.15–0.58) for mothers with higher education [30]. Additionally, a study in Bangladesh observed a modest reduction in ARI symptoms among children of mothers with higher education (OR for fever: 0.809, 95% CI 0.679–0.963) [3]. These divergent results may be attributable to differences in the educational profile of mothers, health literacy, access to health services, and contextual factors such as rurality or healthcare infrastructure. Recognizing the limited and context-dependent impact of maternal education on ARI risk can inform more targeted and effective public health interventions.

In our study, low birth weight was not significantly associated with an increased risk of ARI in children under five, even after multivariate adjustment. This finding is consistent with recent data from Bangladesh, where no significant association was observed between low birth weight and ARI in the adjusted analysis [25]. Other studies, such as those conducted in Zambia, have highlighted the general risk posed by undernutrition and low body weight, but did not specifically examine or report on birth weight as an independent predictor of ARI [30]. The limited exploration of this variable in the literature may be due to challenges in data collection or differences in epidemiological priorities. Nevertheless, our results and the available evidence suggest that low birth weight may not universally predict ARI risk in all settings, underscoring the need for context-specific research to clarify the interplay between early-life nutritional status and susceptibility to respiratory infections. This insight is vital for refining child health interventions and resource allocation in global health agendas.

The lack of significant associations may reflect a combination of factors: (i) the age structure of the sample older under-fives is less proximate to perinatal exposures; (ii) survivor bias, whereby very-low-birthweight or severely undernourished children are underrepresented among older survivors; and (iii) outcome misclassification, because a cough-based definition may capture non-infectious episodes in older ages. Together, these features could attenuate true associations toward the null in design-based models.

First, the outcome was caregiver-reported ARI symptoms over a two-week recall rather than a clinical diagnosis; recognition and recall may vary, especially when the respondent is not the child’s primary attendant, introducing potential recall and classification error. Among children aged ≥2 years, cough episodes from reactive airways disease may be captured by our symptom-based outcome, potentially inflating ARI prevalence in older age bands; any such misclassification is likely non-differential and would bias effect estimates toward the null. Second, the cross-sectional design precludes causal inference and clear temporality (e.g., current sanitation or wealth may not reflect conditions at symptom onset). Third, despite complex-survey adjustment and prespecified confounders, residual confounding by unmeasured environmental exposures (e.g., household air pollution, local viral circulation, day-to-day crowding) may persist. Fourth, immunization in ENDES is abstracted from vaccination cards when available and otherwise from caregiver recall, introducing classification error. Fifth, the age structure of the sample and survivor bias among older under-fives can attenuate associations with early-life risk factors (e.g., very low birth weight, severe undernutrition). Sixth, the two-week recall window and lack of month-specific fieldwork controls may not fully capture seasonality. Finally, categorizing some variables for interpretability can reduce statistical power and mask non-linear patterns. Although ENDES is designed for national and subnational representativeness, differential nonresponse by geography or socioeconomic status may persist; if nonresponse correlates with ARI symptoms, estimates could be biased despite complex-survey adjustment. Complete-case analysis may introduce selection bias if missingness depends on unobserved factors correlated with ARI symptoms (e.g., caregiver time, documentation quality), and the direction of any bias is uncertain.

Taken together, our results provide robust evidence that ARI remains a persistent and complex public health challenge among Peruvian children under five, characterized by both high prevalence and important heterogeneity in risk factors. The lack of significant associations with wealth quintile, maternal education, and low birth weight highlights the need to look beyond traditional social determinants and consider context-specific exposures, health system barriers, and behavioral factors that may drive ARI risk in diverse populations. These findings underscore the importance of using nationally representative data to inform policies and interventions that are both equitable and responsive to local realities. From a global health perspective, the methodological rigor and scope of our study contribute valuable insights for other low- and middle-income countries confronting similar epidemiological patterns. Strengthening targeted prevention strategies such as early detection, community-based education, and tailored vaccination campaigns will be essential to reduce the burden of ARI and close persistent gaps in child health. Ultimately, advancing child survival and achieving international development goals will require coordinated, evidence-based actions informed by the nuanced epidemiology revealed in settings like Peru.

Key strengths include the use of nationally representative data from the 2022 ENDES, a large sample size, and application of complex-survey estimators (weights, strata, PSUs), which enhance accuracy and external validity. The prespecified analytic approach modified Poisson regression to estimate prevalence ratios, and the inclusion of conceptually justified covariates improves the interpretability of independent associations. Despite the noted limitations, the study offers a timely, comprehensive assessment of ARI determinants in a high-relevance national context.

ENDES employs trained interviewers, standardized DHS-aligned modules, and electronic consistency checks and field back-checks, which support internal validity and comparability across regions. Nonetheless, reliance on caregiver report (e.g., ARI symptoms, immunization when cards are unavailable) can introduce recall and classification error; some relevant exposures (e.g., detailed ventilation, cooking location, contact with non-household children, standardized biometrics) are not captured at sufficient granularity for a fully independent assessment. We mitigated these issues by using transparent operational definitions, prespecified covariates informed by theory, and complex-survey estimators; remaining limitations are acknowledged in the Discussion.

From a policy and practice perspective for Peru, our findings support three complementary priorities. First, targeted caregiver education in high-prevalence districts, early recognition of persistent cough, appropriate home care, and prompt care-seeking, together with community-based case finding and clear referral pathways, especially in rural Andes/Amazon settings. Second, routine immunization audit-and-feedback at child contacts (systematic vaccination-card checks, on-the-spot catch-up scheduling, and simple reminders), given the reliance on caregiver recall in ENDES. Third, improvements to the home and service environment, including promotion of improved sanitation, reduction of indoor crowding where feasible, cleaner household fuels to reduce indoor air pollution, and facility readiness for ARI (triage for respiratory complaints, pulse oximetry, and guideline-concordant treatment). To sharpen targeting, subnational risk dashboards built from ENDES and routine data (stratified by sex, age, and region) and behaviorally informed messaging (e.g., Health Belief Model) can help lower practical barriers to timely care-seeking and direct resources to the highest-risk clusters.

Drawing on nationally representative, design-based estimates from ENDES 2022, we recommend a package of pragmatic actions for Peru’s child-health services. First, intensify caregiver education for early recognition of cough and danger signs and prompt care-seeking, with tailored messaging for boys and for children aged 1–5 years, who show higher symptom prevalence. Second, embed routine ARI screening and brief, standardized symptom checks into growth monitoring, vaccination, and sick-child visits, aligned with IMCI triage and referral. Third, strengthen immunization verification and catch-up by systematically reviewing cards during all contacts and closing missed-dose gaps through outreach and reminder/recall. Fourth, scale respiratory-hygiene and handwashing promotion in early-childhood centers and community settings to curb transmission within households and peer groups. Fifth, improve household air quality through coordinated promotion of cleaner cooking and basic ventilation practices in high-risk homes where solid-fuel use persists. Sixth, deliver region-specific outreach that adapts communication and service delivery to natural regions and urban–rural context (e.g., mobile brigades in remote areas), reflecting observed heterogeneity in prevalence. Finally, reinforce care pathways by ensuring pulse oximetry availability, guideline-concordant antibiotics, and referral capacity at first-level facilities, and by instituting equity-focused monitoring of ARI indicators (wealth, maternal education, urban–rural) with attention to seasonal variation.

Several avenues can strengthen the evidence base for child ARI prevention in Peru. First, longitudinal or panel designs that incorporate seasonality and time-varying exposures would clarify temporality and reduce bias inherent to cross-sectional analyses. Second, validation work is needed to refine case definitions, e.g., adding fast breathing or danger signs, or linking with clinical assessments to quantify misclassification and improve specificity beyond cough alone. Third, integrating environmental and contextual data (household fuel type, ventilation proxies, small-area air-quality and weather indicators) would enable more granular attribution of risk. Fourth, methodological improvements, such as survey-weighted multiple imputations for item-missingness, sensitivity analyses for outcome misclassification, and theory-informed confounder selection, can enhance robustness. Fifth, mixed-methods studies with caregivers and frontline providers could illuminate decision-making, care-seeking barriers, and opportunities for tailored messaging. Finally, small-area estimation using pooled DHS/MICS/ENDES waves could map within-country heterogeneity to guide microtargeted interventions and monitoring.

## 5. Conclusions

Using nationally representative 2022 ENDES data and a design-based analytical approach, we found a high two-week prevalence of caregiver-reported ARI symptoms among Peruvian children under five, with systematically higher prevalence in boys and in older age bands relative to infants. These associations remained independent in multivariable modified Poisson models that accounted for sampling weights, stratification, and clustering. Although several social and perinatal factors showed limited or null adjusted associations, these results should be interpreted considering known measurement constraints of household surveys, particularly a cough-based ARI definition, potential non-differential misclassification, and cross-sectional temporality. Taken together, our findings support pragmatic, equity-oriented actions in primary care (targeted caregiver education, routine symptom screening, immunization catch-up) and context-specific outreach across regions and urban–rural settings. By providing transparent, replicable estimates grounded in DHS/ENDES conventions, this study offers immediately usable evidence to refine respiratory-child-health strategies and resource allocation in Peru.

## Figures and Tables

**Table 1:** Sociodemographic and Clinical Characteristics of Children Under Five Years and Their Households According to the 2022 Demographic and Family Health Survey (ENDES), Peru.

Epidemiological Characteristics	n (%)
Child’s Sex	
Female	3100 (49.21)
Male	3199 (50.79)
Current Age	
Under 1 year	2122 (33.69)
1 to <3 years	2207 (35.04)
3 to <5 years	1970 (31.27)
Area of Residence	
Urban	4300 (68.26)
Rural	1999 (31.74)
Wealth Quintile	
1st quintile	1921 (30.50)
2nd quintile	1831 (29.07)
3rd quintile	1194 (18.96)
4th quintile	828 (13.14)
5th quintile	525 (8.33)
Complete National Vaccination Schedule	
No	1949 (30.94)
Yes	4350 (69.06)
Mother’s Educational Level	
No formal education	28 (0.44)
Primary	896 (14.22)
Secondary	3304 (52.46)
Higher	2071 (32.88)
Low Birth Weight	
No	5854(92.94)
Yes	445 (7.06)
Exclusive Breastfeeding	
No	287 (4.56)
Yes	6012 (95.44)
Health Insurance	
No	835 (13.26)
Yes	5464 (86.74)
Acute Respiratory Infection (ARI)	
No	3873 (61.49)
Yes	2426 (38.51)

Source: Authors’ elaboration based on the 2022 Demographic and Family Health Survey (ENDES) database. Note. Percentages are survey-weighted. Standard errors, *p*-values, and 95% confidence intervals (where shown) were estimated using Taylor series linearization under the complex sample design (PSU = v021; strata = v022; pweight = v005/1,000,000). Totals may not sum due to rounding.

**Table 2:** Bivariate Analysis of Sociodemographic and Clinical Characteristics According to the Presence of Acute Respiratory Infection (ARI) among Children Surveyed in ENDES 2022.

Epidemiological Characteristics	Acute Respiratory Infection (ARI)				*p*-Value
	No		Yes		
	(n = 3873)	%	(n = 2426)	%	
Child’s Sex					0.04
Female	1928	49.78	1271	52.39	
Male	1945	50.22	1155	47.61	
Current Age					<0.001
Under 1 year	1384	35.73	738	30.42	
1 to <3 years	1297	33.49	910	37.51	
3 to <5 years	1192	30.78	778	32.07	
Area of Residence					0.29
Urban	2625	67.78	1675	69.04	
Rural	1248	32.22	751	30.96	
Wealth Quintile					0.002
1st quintile	1166	30.11	755	31.12	
2nd quintile	1080	27.89	751	30.96	
3rd quintile	739	19.08	455	18.76	
4th quintile	531	13.71	297	12.24	
5th quintile	357	9.21	168	6.92	
Complete National Vaccination Schedule					0.38
No	1214	31.35	735	30.30	
Yes	2659	68.65	1691	69.70	
Mother’s Educational Level					<0.001
No education	21	0.54	7	0.29	
Primary	531	13.71	365	15.05	
Secondary	1965	50.74	1339	55.19	
Higher	1356	35.01	715	29.47	
Low Birth Weight					0.12
No	3615	93.34	2239	92.29	
Yes	258	6.66	187	7.71	
Exclusive Breastfeeding					0.66
No	180	4.65	107	4.41	
Yes	3693	95.35	2319	95.59	
Health Insurance					0.63
No	3366	86.91	2098	86.48	
Yes	507	13.09	328	13.52	

Source: Authors’ elaboration based on the 2022 Demographic and Family Health Survey (ENDES) database. Note. Percentages are survey-weighted. Standard errors, *p*-values, and 95% confidence intervals (where shown) were estimated using Taylor series linearization under the complex sample design (PSU = v021; strata = v022; pweight = v005/1,000,000). Totals may not sum due to rounding.

**Table 3:** Multivariate Analysis of Factors Associated with Caregiver-Reported ARI Symptoms in Children: Crude and Adjusted Prevalence Ratios from the ENDES 2022 Survey.

Epidemiological Characteristics	Crude PR (95% CI)	*p*-Value	Adjusted PR (95% CI)	*p*-Value
**Child’s Sex**				
Female	Reference		Reference	
Male	1.07 (1.00–1.14)	0.04	1.07 (1.00–1.14)	0.04
**Current Age**				
Under 1 year	Reference		Reference	
1 to <3 years	1.18 (1.09–1.27)	<0.001	1.18 (1.09–1.27)	<0.001
3 to <5 years	1.14 (1.05–1.23)	0.002	1.14 (1.05–1.24)	0.001
**Wealth Quintile**				
1st quintile	Reference		Reference	
2nd quintile	1.04 (0.97–1.13)	0.28	1.06 (0.99–1.15)	0.16
3rd quintile	0.97 (0.89–1.06)	0.51	0.99 (0.91–1.10)	0.98
4th quintile	0.91 (0.82–1.02)	0.09	0.96 (0.86–1.08)	0.53
5th quintile	0.81 (0.71–0.93)	0.003	0.88 (0.76–1.07)	0.08
**Mother’s Educational Level**				
No education	Reference		Reference	
Primary	1.63 (0.85–3.11)	0.14	1.56 (0.82–2.98)	0.18
Secondary	1.62 (0.85–3.08)	0.14	1.55 (0.80–2.97)	0.19
Higher	1.38 (0.73–2.63)	0.33	1.36 (0.71–2.60)	0.35
**Low Birth Weight**				
No	Reference		Reference	
Yes	1.10 (0.98–1.23)	0.11	1.10 (0.98–1.23)	0.09

PR: prevalence ratio; CI 95%: 95% confidence interval. *p*-values were obtained using robust Poisson regression. Source: Authors’ elaboration based on the ENDES 2022 database. Note. Percentages are survey-weighted. Standard errors, *p*-values, and 95% confidence intervals (where shown) were estimated using Taylor series linearization under the complex sample design (PSU = v021; strata = v022; pweight = v005/1,000,000). Totals may not sum due to rounding.

## Data Availability

Publicly available microdata from the 2022 Demographic and Family Health Survey (ENDES) were analyzed in this study. The datasets are freely available from the National Institute of Statistics and Informatics (INEI) public microdata portal; no registration or special permissions are required. No new data were created or analyzed in this study beyond these publicly available files.

## Supplementary Materials

The following supporting information can be downloaded at: https://www.mdpi.com/article/10.3390/children12091242/s1, Table S1: Operational definitions, original DHS/ENDES recode names, recoding rules, and rationale.

## Abbreviations

The following abbreviations are used in this manuscript:

ARI	Acute respiratory infection
CI	Confidence interval
DHS	Demographic and Health Surveys
ENDES	Demographic and Family Health Survey (Peru)
INEI	National Institute of Statistics and Informatics (Peru)
MICS	Multiple Indicator Cluster Surveys
PR	Prevalence ratio
PSU	Primary sampling unit
SDOH	Social Determinants of Health
VIF	Variance inflation factor
