# Supplementary material for: Factors Associated with Acute Respiratory Infections in Children Under Five Years Old: Analysis of the Demographic and Family Health Survey
Source: Children (Basel). 2025 Sep 16;12(9):1242. doi: 10.3390/children12091242 (PMC12468254; doi:10.3390/children12091242)
Supplement: Supplementary file 1 [file children-12-01242-s001.zip › children-3804217-supplementary.pdf]

Supplementary Table S1. Operational definitions, original DHS/ENDES recode names, recoding rules, and rationale.

Variable (Manuscript)	Operational Definition / Recoding Used	Original DHS/ENDES Recode Name(s)
Outcome: ARI symptoms (2-week)	Caregiver-reported cough in the last two weeks; binary (1 = Yes; 0 = No).	H31
Child's sex	Female / Male (as recorded).	B4
Child's age (years)	Under 1; 1 to < 3; 3 to < 5 — derived from age in months.	B8
Area of residence	Urban / Rural.	V102
Household wealth quintile	Wealth index quintiles 1–5 (poorest → wealthiest).	HV270 (household wealth index factor score, quintiles); categories shown with HV271.
Completed national immunization schedule	"Complete" if BCG (H2); 3× DPT/Pentavalent (H3, H5, H7); 3× Polio (H4, H6, H8); and Measles-containing vaccine (H9) recorded on card or caregiver recall; otherwise "No".	S45PV1, S45PV2, S45PV3, S45NM1, S45NM2, S45IF1, S45IF2, S45IF3, S45B0, S45F1, S45F2, S45ML, S45RT1, S45RT2
Mother's educational level	None; Primary; Secondary; Higher.	V106
Low birth weight	< 2,500 g (Yes) vs. ≥ 2,500 g (No) from recorded birthweight.	M19
Exclusive breastfeeding	"Yes" if age 0–5 months, currently breastfeeding, and 24-hour recall shows no other liquids/solids; otherwise "No".	QI440B
Child health insurance	Any public or private child insurance (Yes/No): "Yes" if any insurance item = 1.	S229B1
